# Integrative Single‐Cell and Spatial Transcriptomics Identifies CRIP1^+^ Prehypertrophic Chondrocytes as Inflammatory Hubs and a Valuable Diagnostic Biomarker for Osteoarthritis

**DOI:** 10.1155/mi/1194367

**Published:** 2026-08-25

**Authors:** Zikang Xie, Yu Wang, Jinzhu Liu, He Li, Huwei Bian, Chonghao Gu

**Affiliations:** ^1^ Department of Orthopedics, Changzhou Hospital of Traditional Chinese Medicine, Changzhou Affiliated Hospital of Nanjing University of Chinese Medicine, Changzhou, Jiangsu, China, czzyy.com

**Keywords:** chondrocyte heterogeneity, diagnostic model, machine learning, osteoarthritis, single-cell transcriptomics

## Abstract

Osteoarthritis (OA) is a prevalent degenerative joint disease characterized by progressive cartilage destruction, yet the heterogeneity of transitional chondrocyte states and their contributions to pathogenesis remain incompletely understood. In this study, we constructed a single‐cell transcriptomic atlas of human knee cartilage from 16 OA samples and 2 non‐OA normal donors. High‐resolution sub‐clustering of the prehypertrophic chondrocytes (preHTC) compartment revealed eight transcriptionally distinct subtypes, among which CRIP1^+^ preHTC emerged as the most significantly expanded population in OA, particularly in weight‐bearing regions. Pseudotemporal trajectory inference and regulon analysis identified EGR3 as a candidate key regulator for CRIP1^+^ preHTC. High‐dimensional weighted gene coexpression network analysis (hdWGCNA) demonstrated matrix‐remodeling and proteostasis‐stress transcriptional programs were highly active in CRIP1^+^ preHTC. Cell‐cell communication analysis uncovered that CRIP1^+^ preHTC acts as a central signaling hub in OA, with markedly enhanced FN1 signaling pathway. Spatial transcriptomics confirmed that CRIP1^+^ preHTC co‐localizes with prefibroblasts (preFC) in pathological niches and interacts with other cells in the OA microenvironment. Finally, a machine‐learning‐based 10‐gene classifier (JUN, MCOLN3, RHOC, AKR7A2, HSPG2, AK4, B4GALT2, CLSTN1, LRRC41, and GADD45A) derived from CRIP1^+^ preHTC‐associated hub genes accurately discriminated OA from normal tissues in two independent bulk RNA‐seq cohorts. Collectively, these findings suggest that CRIP1^+^ preHTC may represent a pathogenic cell state in OA, provide a multi‐layered molecular framework that links a specific chondrocyte transitional subset to cartilage degradation, and offer a potentially useful gene signature for OA diagnosis.

## 1. Introduction

Osteoarthritis (OA) is the most prevalent degenerative joint disorder worldwide, affecting hundreds of millions of individuals and imposing a substantial burden on global healthcare systems [1–3]. Pathologically, OA is characterized by progressive degradation of articular cartilage, subchondral bone remodeling, synovial inflammation, and the formation of osteophytes [4, 5]. At the cellular level, the loss of cartilage homeostasis is driven by phenotypic switching and dysregulation of chondrocytes, the sole resident cells within the cartilage matrix [6]. Under pathological conditions such as mechanical overload, aging, and inflammatory insults, chondrocytes undergo hypertrophic differentiation, secrete matrix‐degrading enzymes, and ultimately contribute to extracellular matrix (ECM) breakdown and tissue failure [7, 8]. Despite decades of research, the molecular heterogeneity of chondrocyte populations and the precise transitional states linking homeostasis to pathological hypertrophy remain incompletely understood, limiting the development of disease‐modifying therapies and early diagnostic tools.

Recent advances in single‐cell RNA‐sequencing (scRNA‐seq) have provided unprecedented resolution to dissect cellular heterogeneity in complex tissues, including articular cartilage [9–11]. These technologies have revealed that chondrocytes exist in multiple transcriptionally distinct states, ranging from resting and regulatory subsets to hypertrophic and fibrocartilage‐like populations. Among these, prehypertrophic chondrocytes (preHTC) occupy a critical intermediate position in the differentiation cascade, bridging the gap between healthy mature chondrocytes and terminally differentiated hypertrophic cells [12]. Although hypertrophic chondrocytes (HTC) have been extensively implicated in OA pathogenesis, the preHTC compartment has received comparatively less attention [13]. Existing bulk transcriptomic studies inherently mask the diversity within this population, making it difficult to discern whether preHTC represent a homogeneous transitional state or comprise multiple pathogenic subsets with distinct functional identities. Consequently, the subtype architecture, regulatory networks, spatial organization, and intercellular communication patterns of preHTC in OA cartilage remain largely unexplored.

In this study, we constructed a comprehensive single‐cell transcriptomic atlas of human knee cartilage using 134,551 high‐quality cells from 18 samples (2 non‐OA controls and 16 OA samples). Through high‐resolution subclustering, we dissected the preHTC compartment into eight transcriptionally distinct subtypes and identified CRIP1^+^ preHTC as the most significantly expanded population in OA, particularly in weight‐bearing (WB) regions. Integrative analyses combining pseudotemporal trajectory inference, transcription factor (TF) regulon inference by pySCENIC, high‐dimensional weighted gene coexpression network analysis (hdWGCNA), and cell–cell communication mapping revealed that CRIP1^+^ preHTC exhibits a unique matrix‐remodeling and proteostasis‐stress program, which might be driven by the EGR3 regulon. Furthermore, spatial transcriptomics confirmed that CRIP1^+^ preHTC occupies specific pathological niches and engages in enhanced fibronectin (FN1)‐mediated communication with neighboring fibroblasts (FC) and endothelial cells (EC). Finally, we developed a machine‐learning‐based 10‐gene classifier derived from CRIP1^+^ preHTC‐associated modules that can accurately discriminate OA from normal tissues in independent bulk RNA‐seq cohorts. Together, these findings establish CRIP1^+^ preHTC as a previously unrecognized pathogenic cell state in OA and provide a molecular framework for early diagnosis and potential therapeutic targeting.

Our findings highlight CRIP1^+^ preHTC as a candidate pathogenic hub subset in OA progression and provide a multilayered molecular framework that associates a specific chondrocyte transitional state with cartilage degradation. These results are based on computational analyses of public datasets and should be considered hypothesis‐generating rather than definitive. By translating these single‐cell insights into a clinically applicable gene signature, our work not only deepens the understanding of chondrocyte heterogeneity in joint degeneration but also offers novel insights that may inform future molecular diagnosis and therapeutic development in OA.

## 2. Materials and Methods

### 2.1. Data Acquisition and Sample Information

All datasets used in this study were retrieved from publicly available repositories. The single‐cell RNA‐sequencing (scRNA‐seq) dataset of human knee cartilage was obtained from GSE255460 [9], comprising 18 samples (2 non‐OA normal controls and 16 OA samples), yielding a total of 134,551 high‐quality cells after quality control. Spatial transcriptomics data from OA cartilage sections were obtained from GSM8677818, which was generated using the 10x Genomics Visium platform on cartilage tissues from OA patients. Bulk‐tissue RNA‐seq datasets used for machine‐learning validation included GSE89408, which is derived from synovial tissue (28 normal and 22 OA samples, sequenced on the Illumina HiSeq 2000 platform) as the training cohort [14, 15] and GSE114007, which is derived from articular cartilage (18 normal and 20 OA samples, sequenced on the Illumina HiSeq 2000 platform and Illumina NextSeq 500 platform) as the independent testing cohort [16]. The successful validation of the 10‐gene classifier across different tissue types (synovium and cartilage) highlights the generalizability of the identified OA‐related molecular signature.

### 2.2. Single‐Cell RNA‐Sequencing Data Preprocessing

Single‐cell RNA‐sequencing data were processed using a standardized pipeline using the Seurat R package (v4.4.0). Quality control metrics were applied to filter out low‐quality cells and potential doublets. Data integration across all 18 samples was performed using the Harmony algorithm (v2.0.5) to remove batch effects. Principal component analysis (PCA) was conducted on the integrated gene expression matrix, and the top principal components were selected for uniform manifold approximation and projection (UMAP) visualization. Cell populations were annotated into 11 major lineages based on the expression of established canonical markers: EC, FC, homogeneous chondrocytes (HomC), HTC, interstitial fibrochondrocytes (IntFC), prefibroblasts (preFC), preHTC, preinterstitial fibrochondrocytes (preIntFC), progenitor cells (ProC), regulatory chondrocytes (RegC), and reparative chondrocytes (RepC). Differential expression analysis between clusters was performed using the Wilcoxon rank‐sum test with Bonferroni correction to identify cluster‐specific marker genes, visualized as dot plots and feature plots.

Cellular composition differences between normal and OA cartilages were quantified using stacked bar charts. Tissue preference for each cell type was calculated using the Ro/e (ratio of observed to expected cell number) score [17]. The *R*_*o*_/*e* value quantifies the degree of enrichment or depletion of a given cell type in OA cartilage compared with normal tissue. Specifically, the following thresholds were used to define preference strength: +++ (*R*_*o*_/*e* >1) indicates strong enrichment in tissue; ++ (0.8 < *R*_*o*_/*e* ≤1) represents moderate enrichment; + (0.2 ≤ *R*_*o*_/*e* ≤0.8) denotes weak or neutral preference; ± (0 < *R*_*o*_/*e* <0.2) indicates very weak enrichment or near depletion; and − (*R*_*o*_/*e* = 0) indicates complete absence in tissue. WB and nonweight‐bearing (NWB) regions were further stratified to assess mechanical loading effects on cell distribution.

### 2.3. High‐Resolution Subclustering Analysis

To resolve the heterogeneity within the preHTC compartment, all preHTC cells were extracted and subjected to high‐resolution secondary clustering. Differential gene expression analysis was performed to identify robust subtype‐specific markers. Proportional shifts between normal and OA states were visualized using bar charts and alluvial plots. Kyoto Encyclopedia of Genes and Genomes (KEGG) pathway enrichment analysis was conducted for each subtype to assign functional identities. Absolute infiltration analysis, based on cell type proportions estimated via deconvolution, was performed to statistically evaluate subtype expansion in OA.

### 2.4. Pseudotemporal Trajectory and TF Regulon Analysis

Developmental trajectories of preHTC subtypes were reconstructed using Monocle 2 [18]. Cells were ordered along a pseudotime axis, and branch points were identified to infer fate specification events. Differentially expressed genes along pseudotime were clustered into temporal expression modules, and Gene Ontology Biological Process (GOBP) enrichment analysis was performed for each cluster. Smoothed expression plots were generated to illustrate the dynamic changes in lineage‐specific markers across pseudotime.

TF regulon activity was inferred using pySCENIC [19]. Regulon‐specificity scores (RSS) were calculated to rank the top TFs for each preHTC subtype. Regulon activity was visualized on UMAP embeddings and compared across subtypes to identify master regulators driving subtype‐specific transcriptional programs.

### 2.5. High‐Dimensional Weighted Gene Coexpression Network Analysis (hdWGCNA)

High‐dimensional weighted gene coexpression network analysis (hdWGCNA, v0.4.12) [20] was applied to the preHTC population to identify coexpression modules associated with specific subtypes. Network topology analysis was performed to select an optimal soft threshold power that best fit a scale‐free topology model while maintaining high mean connectivity. Hierarchical clustering of the topological overlap matrix (TOM) was used to define gene modules. Module eigengenes were projected onto the preHTC UMAP to assess spatially restricted activity patterns. Hub genes within each module were identified based on high module membership (kME) and visualized as network plots. Gene Ontology (GO) enrichment analysis was performed for module‐specific gene sets to characterize biological functions.

### 2.6. Cell–Cell Communication Analysis

Intercellular communications and networks were systematically inferred using the CellChat algorithm (v2.1.1) [21]. CellChat analysis was performed to infer relative communication probabilities between cell types by integrating ligand‐receptor expression with prior knowledge of signaling pathways. The inferred communication probabilities were compared descriptively between normal and OA conditions to identify prominent signaling patterns. Ligand–receptor interaction pairs were compared between normal and OA cartilage to identify differential information flow. Circle plots were generated to visualize the number and strength of interactions. Pattern analysis was applied to incoming and outgoing signals to identify dominant communication architectures for each cell subset. Differential pathway activity was assessed to identify enhanced or diminished signaling axes in OA. CellChat analysis was used for pattern recognition and descriptive comparison rather than formal hypothesis testing.

### 2.7. Gene Set Enrichment Analysis (GSEA)

GSEA comparing specific preHTC subtypes against all other preHTC subtypes was performed using hallmark gene sets [22, 23]. Gene set variation analysis (GSVA) was applied across all subtypes to compute pathway activity scores [24].

### 2.8. Spatial Transcriptomics and Microenvironment Mapping

Spatial transcriptomics data from OA cartilage sections (GSM8677818) [25] were analyzed to localize preHTC subtypes within the tissue architecture. Cell‐type deconvolution of spatial spots was performed using RCTD (robust cell‐type decomposition, v1.1) [26] to map the spatial distribution of preHTC subtypes onto tissue sections. Microenvironment dependencies were quantified across three spatial scales: intraspot colocalization (intra), neighborhood within a defined spot radius (juxta), and distal interaction within a larger radius (para). Importance scores were calculated and visualized as heatmaps, and network graphs were constructed to illustrate spatial relationships between specific preHTC subtypes and surrounding cell types.

### 2.9. Machine Learning‐Based Biomarker Discovery and Validation

To develop a diagnostic gene signature for OA, hub genes from selected hdWGCNA modules were extracted as candidate features. Two publicly available bulk RNA‐seq datasets were used: GSE89408 (28 normal, 22 OA) as the training cohort and GSE114007 (18 normal, 20 OA) as the independent testing cohort. Multiple machine‐learning classifiers and combinations were trained, and the model which shows the highest concordance index (C‐index) across both the training and testing cohort was selected as the final model, yielding a multigene signature. In addition, SHAP (SHapley Additive exPlanations) analysis was performed to quantify the contribution of each selected gene to the OA prediction. Model performance was evaluated by receiver operating characteristic (ROC) curve analysis with area under the curve (AUC) and 95% confidence interval (CI) calculation. Confusion matrices were generated to compute accuracy, sensitivity, and specificity in both the training and testing sets.

### 2.10. Statistical Analysis

All statistical analyses were performed using R (version 4.2.1). Normality and homoscedasticity were examined using the Shapiro–Wilk test and Levene’s test, respectively. Parametric tests (*t*‐test and ANOVA) were used for data meeting both standards; otherwise, nonparametric alternatives (Mann–Whitney *U*, Wilcoxon, and Kruskal–Wallis) were applied. A two‐sided *p*‐value <0.05 was considered significant.

## 3. Results

### 3.1. Single‐Cell Transcriptional Landscape of Knee Cartilage in OA Patients

**Figure 1:**
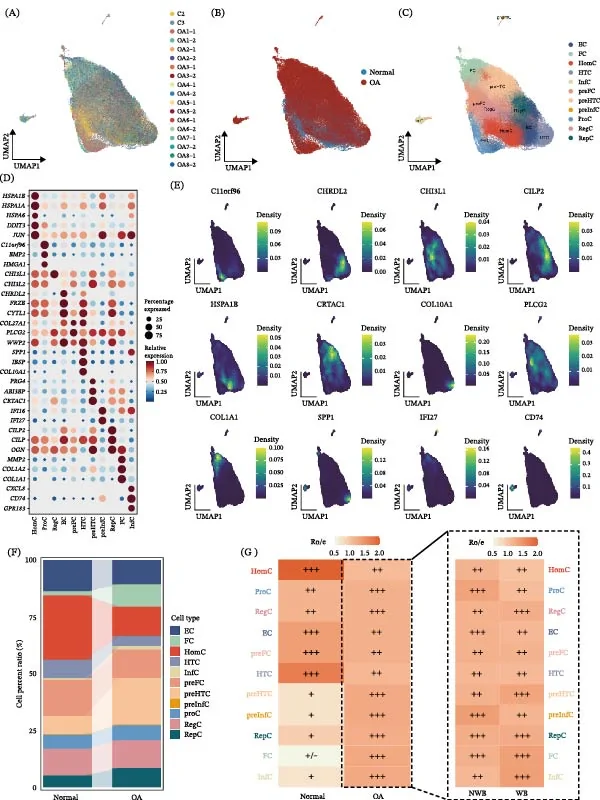
Single‐cell transcriptional landscape of human knee cartilage in osteoarthritis. (A) UMAP visualization of 134,551 high‐quality cells from 18 samples (2 non‐OA and 16 OA) after integration, showing mixing across individuals and tissue types, indicating successful batch effect removal. (B) Separate UMAP plots for non‐OA and OA samples demonstrate comparable embedding structures and cellular distributions. (C) All cells are classified into 11 major cell types based on lineage markers: endothelial cells (EC), fibroblasts (FC), homogeneous chondrocytes (HomC), hypertrophic chondrocytes (HTC), interstitial fibrochondrocytes (IntFC), prefibroblasts (preFC), prehypertrophic chondrocytes (preHTC), preinterstitial fibrochondrocytes (preIntFC), progenitor cells (ProC), regulatory chondrocytes (RegC), and reparative chondrocytes (RepC). (D) Dot plot confirms that each cell population expresses its expected identity genes, such as CHRDL2 and FRZB for EC, COL1A1, COL1A2, and MMP2 for FC, and COL10A1 and IBSP for HTC. (E) Feature plots validate the spatial distribution of canonical markers across the UMAP embedding for selected cell types. (F) Stacked bar chart comparing cellular composition between normal and OA cartilage reveals a marked remodeling of the microenvironment. (G) Tissue preference quantification (*R*_*o*_/*e* score) shows that preHTC, preFC, and IntFC are enriched in OA cartilage (*R*_*o*_/*e* > 1, left panel), and stratification by weight‐bearing (WB) status demonstrates that preHTC accumulation is pronounced in WB OA regions compared with nonweight‐bearing (NWB) areas (right panel).

We first constructed a single‐cell atlas of human knee cartilage using 134,551 high‐quality cells from 18 samples (2 non‐OA and 16 OA samples). Following data integration, the UMAP visualization showed that cells from different tissue types and individuals mixed well across the embedding, indicating successful removal of batch effects (Figure 1A,B). Based on the expression of established lineage markers, all cells were categorized into 11 major celltypes: EC, FC, HomC, HTC, IntFC, preFC, preHTC, preIntFC, ProC, RegC, and RepC (Figure 1C). The dot plot confirmed that each population carried expected identity genes; for instance, EC expressed CHRDL2 and FRZB, FC showed high COL1A1, COL1A2, and MMP2, and HTC were marked by COL10A1 and IBSP (Figuer 1D). Feature plots further validated the spatial distribution of these canonical markers across the UMAP embedding (Figure 1E).

When we compared cellular composition between normal and OA cartilage, the stacked bar chart revealed a marked remodeling of the microenvironment. FC and preHTC proportions are increased substantially in OA, whereas HomC and HTC proportions are significantly decreased (Figure 1F). Because FCs represent terminal executors of pathological fibrosis, whereas preHTCs occupy an upstream position in the differentiation cascade and may reflect early disease‐driven reprogramming, we decided to focus on preHTC for further investigation. Tissue preference quantification using the Ro/e score confirmed that preHTC, preFC, and IntFC were enriched in OA cartilage (Ro/e >1), while HomC and RepC showed strong normal tissue preference (Figure 1G, left). Stratification by WB status further showed that preHTC accumulation was pronounced in WB OA regions compared with NWB areas, suggesting that mechanical loading influences the distribution of this population (Figure 1G, right).

### 3.2. Transcriptional Heterogeneity and Plasticity of preHTC Subpopulations

**Figure 2:**
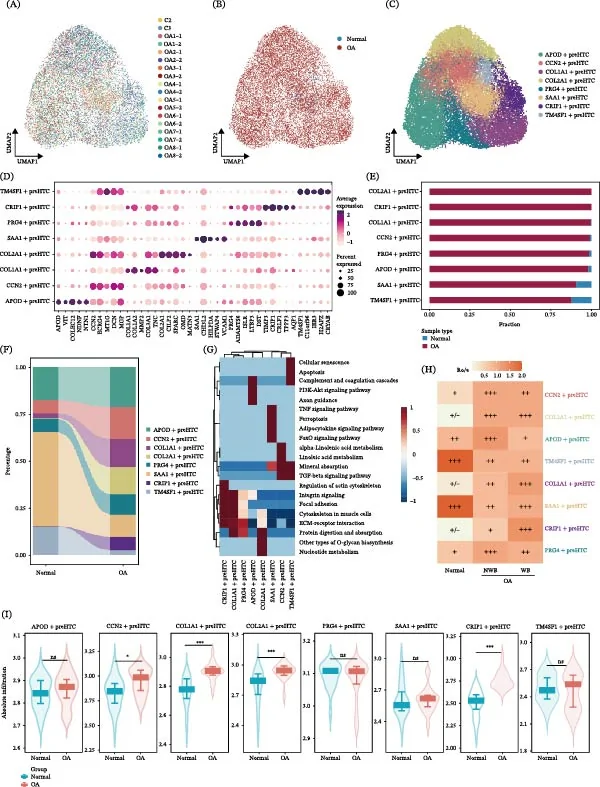
Transcriptional heterogeneity and plasticity of preHTC subpopulations. (A) High‐resolution secondary clustering after extracting 24,888 preHTC cells from the full dataset (colored by different samples). (B) UMAP plot of the 24,888 preHTC cells colored by sources from normal or OA. (C) Separate UMAP plots for each of the eight preHTC subtypes: APOD^+^, CCN2^+^, COL1A1^+^, COL2A1^+^, PRG4^+^, SAA1^+^, CRIP1^+^, and TM4SF1^+^ preHTC. (D) Differential expression analysis identifies robust marker genes for each cluster, with APOD^+^ preHTC highly expressing APOD and VIT, CCN2^+^ preHTC expressing CCN2 and DCN, COL2A1^+^ preHTC retaining COL2A1, and CRIP1^+^ preHTC distinguished by CRIP1 and TIMP3. (E) Bar chart of sample‐type composition shows that the relative abundance of several subpopulations shifts markedly between normal and OA tissues. (F) Alluvial plot illustrates proportional changes across disease states, revealing that COL1A1^+^ and CRIP1^+^ preHTC expand in OA while SAA1^+^ and TM4SF1^+^ preHTC contract. (G) KEGG pathway enrichment analysis assigns distinct functional identities to each subtype, with ECM‐receptor interaction and focal adhesion prominent in COL1A1^+^ and CRIP1^+^ preHTC, and inflammatory pathways such as TNF signaling and ferroptosis enriched in SAA1^+^ preHTC. (H) Tissue‐preference analysis across NWB and WB regions reveals that CRIP1^+^ and COL1A1^+^ preHTC strongly favor WB areas, whereas APOD^+^ and PRG4^+^ preHTC are more evenly distributed. Specifically, +++ (*R*_*o*_/*e* >1) indicates strong enrichment in tissue; ++ (0.8 < *R*_*o*_/*e* ≤1) represents moderate enrichment; + (0.2 ≤ *R*_*o*_/*e* ≤0.8) denotes weak or neutral preference; +/− (0 < *R*_*o*_/*e* <0.2) indicates very weak enrichment or near depletion in tissue. (I) Absolute infiltration analysis identifies CRIP1^+^ preHTC as the most significantly expanded subtype in OA (*p* < 0.001). Significance was determined by Wilcoxon rank‐sum test with Benjamini–Hochberg correction;  ^∗^ *p* < 0.05;  ^∗∗∗^ *p* < 0.001; ns, not significant.

To dissect the internal structure of the preHTC compartment, we extracted 24,888 preHTC cells and performed high‐resolution secondary clustering (Figure 2A,B). Subsequently, eight distinct subtypes were identified: APOD^+^ preHTC, CCN2^+^ preHTC, COL1A1^+^ preHTC, COL2A1^+^ preHTC, PRG4^+^ preHTC, SAA1^+^ preHTC, CRIP1^+^ preHTC, and TM4SF1^+^ preHTC (Figure 2C). Differential expression analysis identified robust marker genes for each cluster. APOD^+^ preHTC highly expressed APOD and VIT; CCN2^+^ preHTC showed strong CCN2 and DCN; COL2A1^+^ preHTC retained classical cartilage markers such as COL2A1; and CRIP1^+^ preHTC was distinguished by CRIP1 and TIMP3 (Figure 2D). The bar chart of sample‐type composition indicated that the relative abundance of some subpopulations shifted markedly between normal and OA tissues (Figure 2E). The alluvial plot illustrated these proportional changes across disease states, showing that COL1A1^+^ and CRIP1^+^ preHTC expanded in OA, while SAA1^+^ and TM4SF1^+^ preHTC contracted (Figure 2F).

KEGG pathway enrichment analysis assigned distinct functional identities to each subtype. ECM‐receptor interaction and focal adhesion were prominent in COL1A1^+^ and CRIP1^+^ preHTC, whereas inflammatory pathways such as TNF signaling and Ferroptosis were enriched in SAA1^+^ preHTC (Figure 2G). Tissue preference analysis across NWB and WB regions revealed that CRIP1^+^ and COL1A1^+^ preHTC strongly favored WB areas, whereas APOD^+^ and PRG4^+^ preHTC were more evenly distributed (Figure 2H). Notably, absolute infiltration analysis identified CRIP1^+^ preHTC as the most significantly expanded subtype in OA (*p* < 0.001), whereas several other subtypes showed modest or nonsignificant changes (Figure 2I). These observations directed our subsequent focus toward CRIP1^+^ preHTC as a population of interest that shows the most prominent expansion in OA progression.

### 3.3. Pseudotemporal Ordering and Transcription‐Factor Regulons in preHTC

We next used Monocle 2 to reconstruct the developmental trajectory of preHTC subtypes. The resulting graph displayed a continuous pseudotime axis with multiple branch points, suggesting that preHTC subpopulations arise from a common progenitor pool and undergo gradual fate specification (Figure 3A). Pseudotime values progressed from dark blue (early) to light blue (late) along the trajectory (Figure 3B), and state assignment partitioned cells into seven distinct states that partially overlapped with subtype labels (Figure 3C). Heatmap clustering of differentially expressed genes along pseudotime identified four major gene clusters with distinct temporal dynamics. Genes in cluster 1 (e.g., COL2A1 and PRG4) were highest in early pseudotime and declined thereafter, whereas cluster 3 genes (e.g., COL1A1, ANXA1, and PTMA) showed the opposite trend. GOBP analysis linked these clusters to ECM organization, cell differentiation, and the inflammatory response (Figure 3D). Smoothed expression plots further illustrated how representative markers of each subtype evolved across pseudotime; for example, COL2A1 decreased as COL1A1 and SAA1 increased, consistent with a shift from resting toward an activated, matrix‐remodeling phenotype (Figure 3E).

Figure 3Pseudotemporal ordering and transcription‐factor regulons in preHTC. (A) Developmental trajectory of preHTC subtypes reconstructed using Monocle 2, showing a continuous pseudotime axis with multiple branch points, suggesting that preHTC subpopulations arise from a common progenitor pool and undergo gradual fate specification. (B) Pseudotime values progress from dark blue (early) to light blue (late) along the trajectory. (C) State assignment partitions cells into seven distinct states that partially overlap with subtype labels. (D) Heatmap clustering of differentially expressed genes along pseudotime identifies four major gene clusters with distinct temporal dynamics, and GOBP analysis links these clusters to extracellular matrix organization, cell differentiation, and inflammatory response. (E) Smoothed expression plots illustrate how representative markers of each subtype evolve across pseudotime, with COL2A1 decreasing as COL1A1 and SAA1 increase, consistent with a shift from a resting toward an activated, matrix‐remodeling phenotype. (F) Regulon activity heatmap inferred by pySCENIC shows clear differential activity across the eight subtypes. (G) Regulon‐specificity scoring ranks the top transcription factors for each cluster. (H) UMAP plot shows that EGR3 regulon activity is concentrated in the CRIP1^+^ preHTC region. (I) Violin plot comparison confirms that EGR3 regulon activity is significantly higher in CRIP1^+^ preHTC than in all other subtype.

**Figure:**
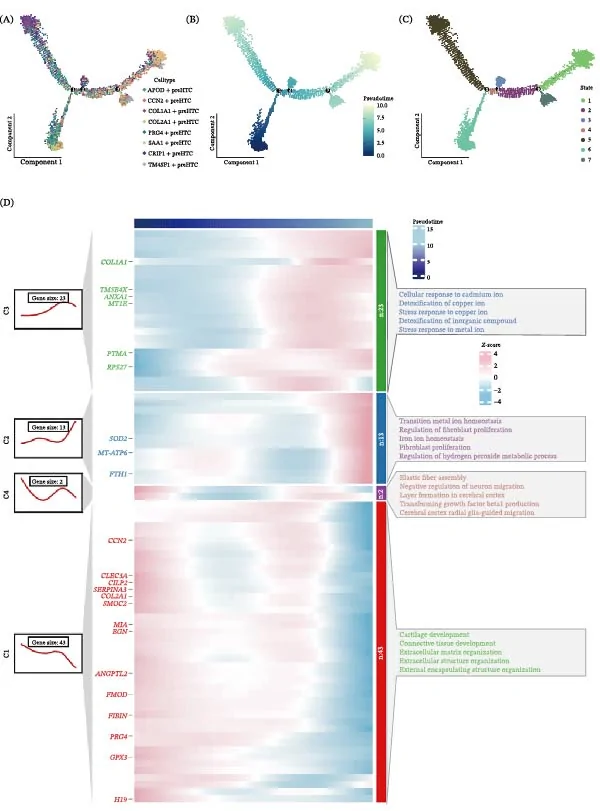

**Figure:**
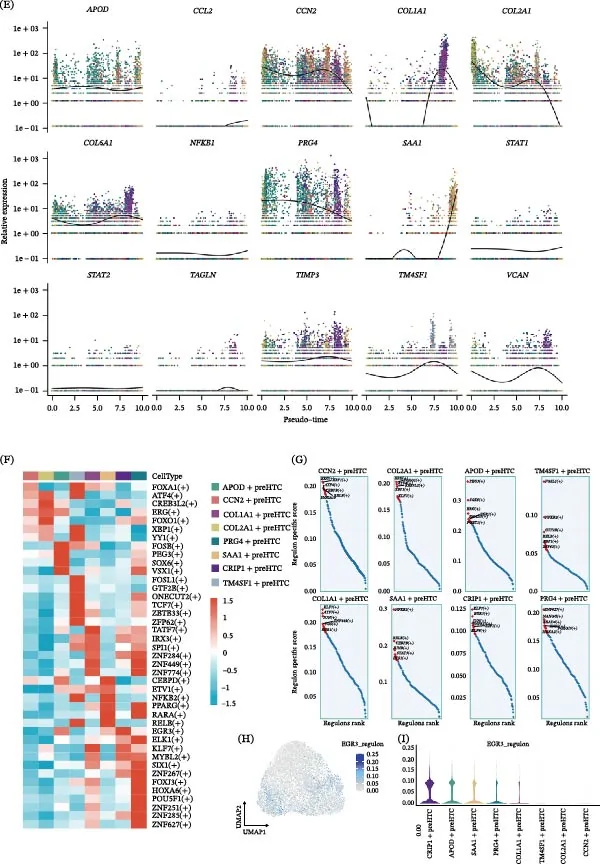

To uncover the regulatory network underlying these transitions, we inferred TF regulon activity using pySCENIC. The regulon activity heatmap showed clear differential activity across the eight subtypes (Figure 3F). Regulon‐specificity scoring ranked the top TFs for each cluster (Figure 3G). Among these, EGR3 emerged as a particularly strong candidate because its regulon activity was concentrated in CRIP1^+^ preHTC on the UMAP plot (Figure 3H) and was significantly higher in this subtype than in all others by violin plot comparison (Figure 3I). These data suggest that EGR3‐driven transcriptional programs may confer the specific functional identity of CRIP1^+^ preHTC in OA cartilage.

### 3.4. hdWGCNA Identifies CRIP1^+^ preHTC‐Associated Gene Coexpression Modules

We applied hdWGCNA to the preHTC dataset to identify coexpression modules that correlate with specific subtypes. Network topology analysis indicated that a soft threshold power of 8 produced the best fit to a scale‐free topology model while maintaining high mean connectivity (Figure 4A). Hierarchical clustering of the TOM yielded 10 distinct gene modules, labeled M1 through M10, and color‐coded for downstream analysis (Figure 4B). Each module contained a characteristic set of genes ranked by module membership (kME); for example, M6 included COPG1, CKAP4, and PKM, whereas M8 contained EIF4A1, SLC39A1, and HSP90AA1 (Figure 4C).

Figure 4hdWGCNA identifies CRIP1^+^ preHTC‐associated gene coexpression modules and its biological functions. (A) Network topology analysis indicates that a soft threshold power of eight produces the best fit to a scale‐free topology model while maintaining high mean connectivity. (B) Hierarchical clustering of the topological overlap matrix yields 10 distinct gene modules (M1–M10), color‐coded for downstream analysis. (C) Each module contains a characteristic set of genes ranked by module membership (kME). (D) Projection of module eigengenes onto the preHTC UMAP reveals spatially restricted activity patterns. (E) Global visualization of hub gene networks illustrates the internal connectivity architecture for all 10 modules. (F) Module correlation matrix shows moderate positive relationships among several modules but relative independence of M6 and M8 from the others. (G) Dot plot of module gene expression confirms that M6 and M8 are selectively enriched in CRIP1^+^ preHTC, with high average expression and high percent expression in this subtype. (H) Network plot of M6 hub genes highlights a tightly connected gene set centered on matrix regulators. (I) Network plot of M8 hub genes highlights a tightly connected gene set centered on stress‐response genes. (J,K) GO enrichment analysis shows that (J) M6 genes are strongly enriched in extracellular matrix organization, extracellular structure organization, and collagen fibril organization, (K) and M8 genes are enriched in response to unfolded protein, response to topologically incorrect protein, and chaperone‐mediated protein folding.

**Figure:**
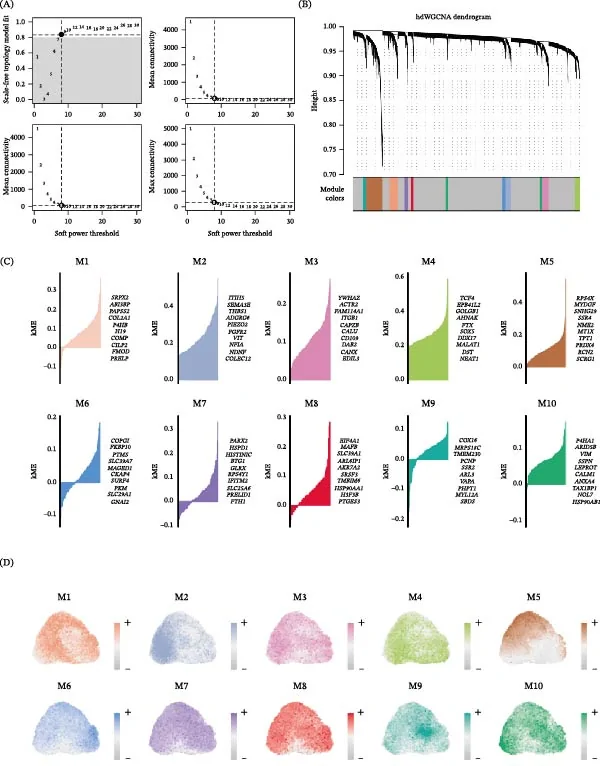

**Figure:**
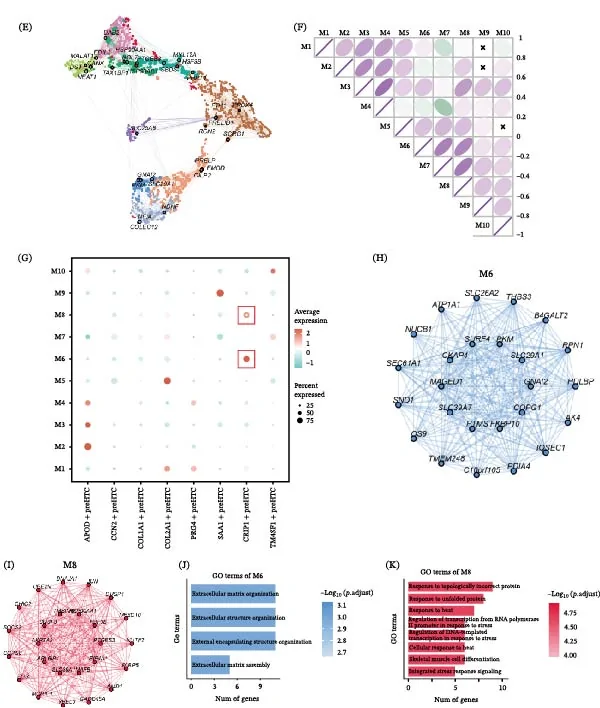

Projection of module eigengenes onto the preHTC UMAP revealed spatially restricted activity patterns. M6 and M8 signals were concentrated in the CRIP1^+^ preHTC region, whereas M3 and M4 mapped preferentially to APOD^+^ preHTC (Figure 4D). Global visualization of hub gene networks illustrated the internal connectivity architecture for all 10 modules (Figure 4E), and the module correlation matrix showed moderate positive relationships among several modules but relative independence of M6 and M8 from the others (Figure 4F). The dot plot of module gene expression confirmed that M6 and M8 were selectively enriched in CRIP1^+^ preHTC, with high average expression and high percent expression in this subtype (Figure 4G). Network plots of M6 and M8 hub genes highlighted tightly connected gene sets centered on matrix regulators (M6) and stress‐response genes (M8) (Figure 4H,I). Consistent with these architectures, GO enrichment analysis showed that M6 genes were strongly enriched in ECM organization, extracellular structure organization, and collagen fibril organization (Figure 4J), whereas M8 genes were enriched in response to unfolded protein, response to topologically incorrect protein, and chaperone‐mediated protein folding (Figure 4K). Together, these modules suggest that a matrix‐remodeling and proteostasis‐stress program is specifically active in CRIP1^+^ preHTC.

### 3.5. Cell–Cell Communication Networks are Restructured Around CRIP1^+^ preHTC in OA

CellChat was used to systematically compare intercellular communication between normal and OA cartilages. Circle plots of interaction number and strength showed that OA cartilage harbored more numerous and stronger ligand‐receptor connections than normal tissue (Figure 5A). Pattern analysis of incoming signals revealed that OA chondrocyte subsets received altered communication architectures, with certain preHTC subtypes gaining new dominant inputs (Figure 5B, top). Similarly, outgoing signal patterns from secreting cells were restructured in OA, indicating a global rewiring of the cartilage signaling network (Figure 5B, bottom).

Figure 5Cell–cell communication networks are inferred around CRIP1^+^ preHTC in OA. (A) Circle plots of interaction number and strength show that OA cartilage harbors more numerous and stronger ligand‐receptor connections than normal tissue. (B) Pattern analysis of incoming signals (top) reveals that OA chondrocyte subsets receive altered communication architectures, and outgoing signal patterns (bottom) are also restructured, indicating global rewiring of the cartilage signaling network. (C) Differential information flow analysis identifies enhanced signaling through MIF, CCL, CXCL, and ICAM pathways in OA, whereas several homeostatic pathways are diminished. (D) Focusing on CRIP1^+^ preHTC as a sender cell, this subtype engages distinct ligand‐receptor pairs with recipient cells in OA compared with normal tissue. (E) In normal cartilage, FN1‐mediated communication between CRIP1^+^ preHTC and partner cells is weak. (F) In OA, CRIP1^+^ preHTC develops FN1 signaling connections with EC, FC, HTC, and multiple other preHTC subtypes, with markedly elevated communication probability.

**Figure:**
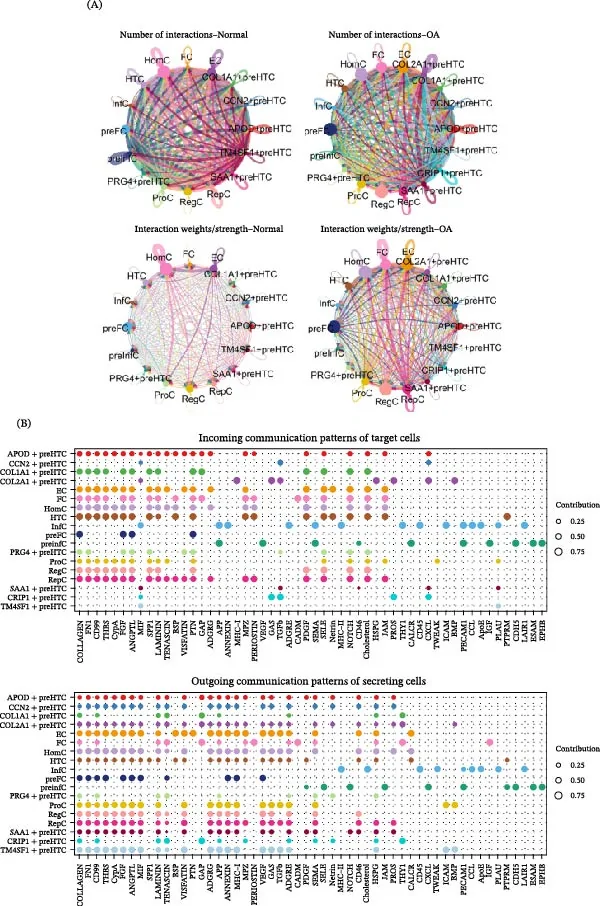

**Figure:**
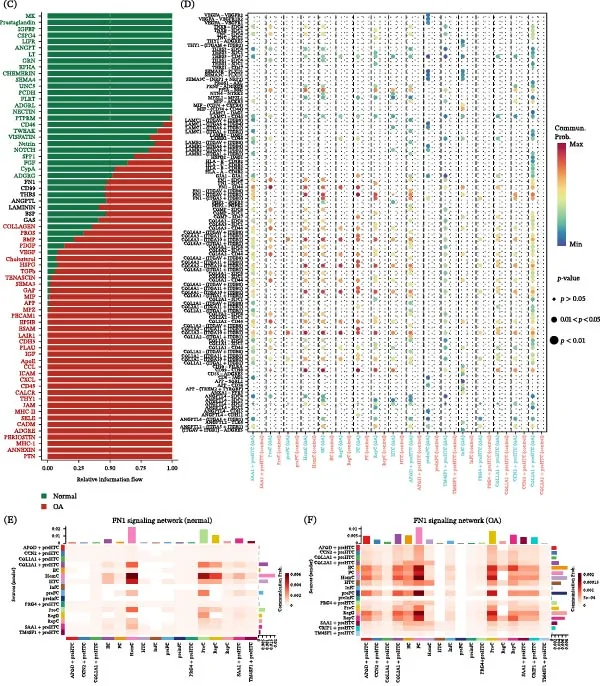

Differential information flow analysis identified multiple pathways with altered activity. OA cartilage showed enhanced signaling through MIF, CCL, CXCL, and ICAM pathways, whereas several homeostatic pathways were diminished (Figure 5C). Focusing on CRIP1^+^ preHTC as a sender cell, we observed that this subtype engaged distinct ligand‐receptor pairs with recipient cells in OA compared with normal tissue (Figure 5D). The FN1 (fibronectin) signaling network emerged as one of the most prominent OA‐specific axes. In normal cartilage, FN1‐mediated communication between CRIP1^+^ preHTC and partner cells was weak (Figure 5E). In OA, CRIP1^+^ preHTC developed FN1 signaling connections with EC, FC, HTC, and multiple other preHTC subtypes, with markedly elevated inferred communication probability compared to normal cartilage (Figure 5F). Further examination of the top‐ranked ligand‐receptor pairs revealed that this enhanced FN1 signaling was primarily mediated through the FN1–ITGA5/ITGAV/ITGB1 (integrin receptor complexes) and FN1–SDC1/SDC2 (syndecan receptors) axes, which showed higher communication probabilities in OA than in normal tissues. These observations are consistent with the known pro‐inflammatory and matrix‐remodeling roles of FN1 in OA pathogenesis.

### 3.6. Spatial Localization and Microenvironment Dependencies of preHTC Subtypes

**Figure 6:**
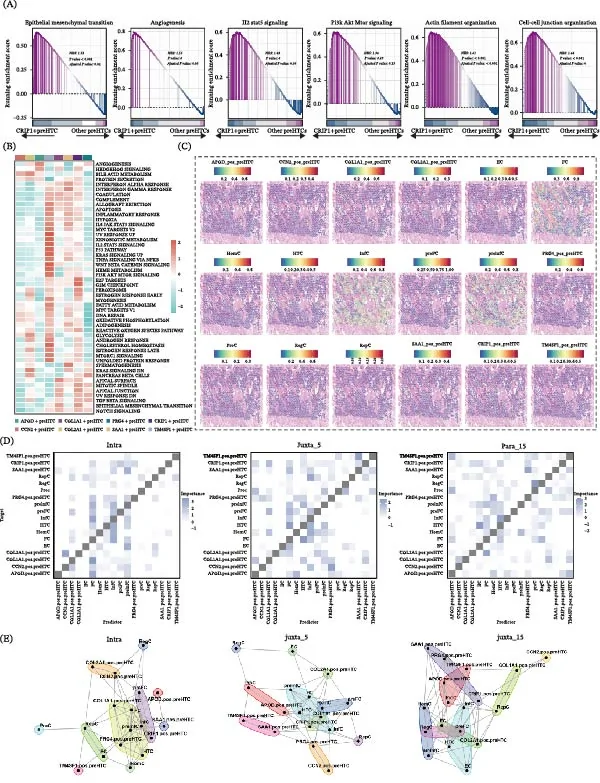
Spatial localization and microenvironment dependencies of preHTC subtypes. (A) GSEA comparing CRIP1^+^ preHTC against all other preHTC subtypes reveals significant enrichment in epithelial‐mesenchymal transition, angiogenesis, PI3K‐Akt‐mTOR signaling, actin filament organization, and cell–cell junction organization. (B) Hallmark GSVA across all eight subtypes confirms that CRIP1^+^ preHTC carries a high proliferative activity, whereas PRG4^+^ preHTC retains signatures of normal cartilage homeostasis. (C) SpatialFeaturePlot shows the spatial distribution of each cell type mapped onto tissue sections using RCTD deconvolution, with preHTC subtypes occupying distinct zones within the cartilage matrix. (D) Importance score heatmaps show scale‐specific association patterns across three spatial scales (intraspot colocalization, juxta_5, and para_15); for instance, CRIP1^+^ preHTC exhibits strong intrascale colocalization with preFC. (E) Network graphs visualize these relationships, showing that CRIP1^+^ preHTC occupies a central position in the local microenvironment network with thick edges connecting it to matrix‐producing and inflammatory subtypes at the intra scale.

To translate these findings into a spatial context, we analyzed spatial transcriptomics data from OA cartilage sections. GSEA comparing CRIP1^+^ preHTC against all other preHTC subtypes revealed significant enrichment in epithelial‐mesenchymal transition, angiogenesis, PI3K‐Akt‐mTOR signaling, actin filament organization, and cell–cell junction organization (Figure 6A). Hallmark GSVA across all eight subtypes confirmed that CRIP1^+^ preHTC carried a high proliferative activity, whereas PRG4^+^ preHTC retained signatures of normal cartilage homeostasis (Figure 6B).

Using RCTD deconvolution, we mapped the spatial distribution of each cell type onto tissue sections. The SpatialFeaturePlot showed that preHTC subtypes occupied distinct zones within the cartilage matrix (Figure 6C). We then quantified microenvironment dependencies across three spatial scales: intraspot colocalization (intra), neighborhood within a 5‐spot radius (juxta_5), and distal interaction within a 15‐spot radius (para_15). Importance score heatmaps showed scale‐specific association patterns; for instance, CRIP1^+^ preHTC exhibited strong intrascale colocalization with preFC (Figure 6D). Network graphs visualized these relationships, showing that CRIP1^+^ preHTC occupied a central position in the local microenvironment network, with thick edges connecting it to matrix‐producing and inflammatory subtypes at the intra scale (Figure 6E). These spatial data demonstrate that CRIP1^+^ preHTC acts as a pathological hub that coordinates local matrix destruction in OA cartilage.

### 3.7. A Gene Signature Classifier Derived From CRIP1^+^ preHTC was Established to Discriminate OA

**Figure 7:**
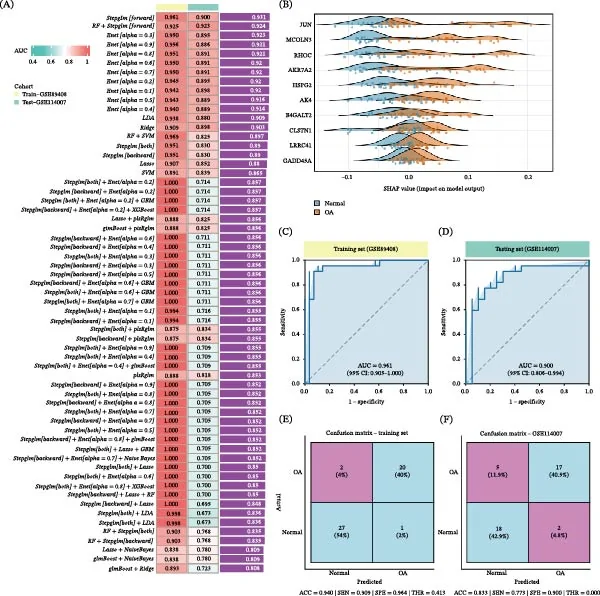
A gene signature classifier is established derived from CRIP1^+^ preHTC to accurately discriminate OA from normal cartilage. (A) Multiple machine‐learning classifiers are trained using GSE89408 as the training cohort and GSE114007 as the independent testing cohort; the Stepglm[forward] model achieves the highest average concordance index (C‐index) across both cohorts. (B) SHAP value analysis shows that JUN, MCOLN3, and RHOC contribute most strongly to OA prediction, followed by AKR7A2 and HSPG2. (C) ROC curve in the training set (GSE89408) yields an AUC of 0.961 (95% CI: 0.905–1.000). (D) ROC curve in the independent testing set (GSE114007) yields an AUC of 0.900 (95% CI: 0.806–0.994). (E) Confusion matrix analysis in the training set shows an overall accuracy of 0.940, with sensitivity 0.909 and specificity 0.964. (F) Confusion matrix analysis in the testing set shows an overall accuracy of 0.833, with sensitivity 0.773 and specificity 0.900.

Finally, we tested whether the molecular programs active in CRIP1^+^ preHTC could serve as potential diagnostic biomarkers. We extracted hub genes from the hdWGCNA modules M6 and M8 and trained multiple machine‐learning classifiers to distinguish OA from normal cartilage using GSE89408 (28 normal, 22 OA) as the training cohort and GSE114007 (18 normal, 20 OA) as the independent testing cohort. The Stepglm[forward] model achieved the highest average concordance index (C‐index) across both cohorts (Figure 7A). This model selected a 10‐gene signature comprising JUN, MCOLN3, RHOC, AKR7A2, HSPG2, AK4, B4GALT2, CLSTN1, LRRC41, and GADD45A. SHAP value analysis showed that JUN, MCOLN3, and RHOC contributed most strongly to the OA prediction, followed by AKR7A2 and HSPG2 (Figure 7B).

Performance evaluation confirmed robust discriminatory capacity. In the training set (GSE89408), the ROC curve yielded an AUC of 0.961 (95% CI: 0.905–1.000) (Figure 7C). In the independent testing set (GSE114007), the AUC remained high at 0.900 (95% CI: 0.806–0.994) (Figure 7D). Confusion matrix analysis showed that the classifier achieved an overall accuracy of 0.940 in the training set, with sensitivity 0.909 and specificity 0.964 (Figure 7E). In the testing set, the accuracy was 0.833, sensitivity 0.773, and specificity 0.900 (Figure 7F). Taken together, these results demonstrate that the transcriptional signature of CRIP1^+^ preHTC, operationalized through a concise 10‐gene classifier, possesses strong potential as a molecular diagnostic tool for OA.

## 4. Discussion

In this study, we constructed a comprehensive single‐cell transcriptomic atlas of human knee cartilage, encompassing 134,551 cells from 18 samples, and dissected the heterogeneity of preHTC in OA pathogenesis. Our analyses revealed that the preHTC compartment is composed of eight transcriptionally distinct subtypes, among which CRIP1^+^ preHTC emerges as the most significantly expanded population in OA cartilage, particularly in WB regions. By integrating pseudotemporal trajectory inference, regulon analysis, high‐dimensional WGCNA, cell–cell communication mapping, spatial transcriptomics, and machine‐learning‐based classifier development, we provide a multilayered molecular framework linking a specific preHTC subset to OA progression and diagnostic biomarker discovery.

While chondrocyte hypertrophy is a well‐recognized feature of OA, the intermediate prehypertrophic stage has received less attention. Our high‐resolution subclustering of preHTC cells identified a previously underappreciated subset marked by CRIP1 and TIMP3 that expands dramatically in OA. The enrichment of this subset in weight‐bearing regions is suggestive of a potential role for mechanical loading in its accumulation, consistent with the known role of biomechanical stress in OA pathogenesis. KEGG analysis showed that CRIP1^+^ preHTC is enriched in ECM‐receptor interaction and focal adhesion pathways, aligning with the idea that these cells actively remodel the cartilage matrix. Moreover, the coexpression modules M6 and M8, which are specifically active in CRIP1^+^ preHTC, are enriched in ECM organization and unfolded protein response, respectively. The concurrent activation of matrix‐remodeling and proteostasis‐stress programs may reflect a maladaptive secretory phenotype that contributes to cartilage degradation.

The pseudotime trajectory indicated that preHTC subtypes follow a continuous differentiation path, with CRIP1^+^ preHTC occupying a distinct branch. This aligns with the concept that OA‐associated chondrocytes undergo phenotypic switching rather than simply entering terminal hypertrophy. Our regulon analysis identified EGR3 as a candidate master regulator in CRIP1^+^ preHTC. EGR3 has been implicated in inflammatory responses and tissue remodeling in other systems [27–29], but its role in OA chondrocytes has not been described. The selective activity of EGR3 regulon in this subset raises the possibility that it may orchestrate the expression of matrix‐degrading enzymes and stress‐response genes. Future functional studies using chondrocyte‐specific Egr3 deletion or overexpression will be required to rigorously test this hypothesis.

At the intercellular communication level, CRIP1^+^ preHTC becomes a central signaling hub in OA, with enhanced FN1‐mediated interactions with FC, EC, and other chondrocyte subsets. Fibronectin is a known damage‐associated molecular pattern that can stimulate inflammatory and catabolic responses in joint tissues [29–31]. The increased FN1 signaling originating from CRIP1^+^ preHTC may therefore propagate a procatabolic cascade throughout the cartilage microenvironment. Targeting FN1 or its receptors might offer a potential avenue for interrupting this pathological crosstalk.

The spatial transcriptomics analysis provided direct evidence that CRIP1^+^ preHTC resides in specific niches and exhibits strong colocalization with preFC. This spatial proximity suggests that these two cell types may cooperate in driving fibrosis and matrix stiffening. The network analysis at three spatial scales (intraspot, juxta, and para) revealed that CRIP1^+^ preHTC has both immediate and short‐range influences on neighboring cells, reinforcing its role as a local orchestrator of OA pathology.

A clinically useful finding of this study is the development of a 10‐gene classifier derived from CRIP1^+^ preHTC‐associated modules. The classifier achieved excellent discriminatory performance in both training (AUC = 0.961) and independent validation (AUC = 0.900) cohorts, outperforming many previously reported OA biomarkers. The signature includes genes such as JUN, MCOLN3, and RHOC, which have been linked to cartilage degradation, autophagy, and cytoskeletal remodeling, respectively. The ability to distinguish OA from normal cartilage using bulk RNA‐seq data from synovial or cartilage biopsies suggests that this signature may serve as a candidate diagnostic tool, warranting further validation in prospective cohorts. However, further validation in large, multicenter cohorts and in easily accessible samples (e.g., peripheral blood or urine) will be necessary before clinical application.

Several limitations should be acknowledged. First, although the single‐cell data were derived from 16 OA samples, the normal control group included only two non‐OA donors, which may limit the generalizability of normal‐vs‐OA comparisons. Second, the inference of regulatory networks (pySCENIC) and cell–cell communication (CellChat) is computational and requires experimental validation such as immunohistochemical costaining for CRIP1/TIMP3/EGR3, FN1‐integrin functional blockade in cartilage explants, and CRIP1 knockdown assays in chondrocyte models. Third, the diagnostic model was built on bulk RNA‐seq data from publicly available cohorts that have relatively small sample sizes; prospective validation in a dedicated patient cohort is needed. Fourth, the functional role of CRIP1 itself in chondrocyte biology remains unexplored. Future studies should investigate whether CRIP1 is merely a marker or actively contributes to the pathogenic phenotype of this preHTC subset.

In summary, this work provides a high‐resolution molecular dissection of preHTCs in OA and identifies CRIP1^+^ preHTC as a candidate pathogenic subset that shows expansion in OA, exhibits a distinct matrix‐remodeling and stress‐response program in the analyzed dataset, demonstrates altered intercellular communication via FN1 signaling, and shows spatial proximity with pre‐FC. Furthermore, a compact gene signature derived from this subset shows strong potential as a candidate diagnostic tool and requires further validation. These findings highlight potential directions for understanding OA pathogenesis and could guide future research toward diagnostic and therapeutic applications.

## 5. Conclusion

Our study provides a high‐resolution single‐cell and spatial transcriptomic atlas of human knee OA and reveals that the preHTC compartment in OA is heterogeneous and consists of distinct subtypes. Among these, CRIP1^+^ preHTC emerges as a previously unrecognized pathogenic subset that expands markedly in OA. CRIP1^+^ preHTC is characterized by a unique matrix‐remodeling and proteostasis‐stress program, which is potentially regulated by the TF EGR3, and acts as a central signaling hub through enhanced FN1‐mediated intercellular communication. Spatial transcriptomics further confirms its colocalization with preFC and other cell types in pathological niches. A machine‐learning‐based gene classifier was established from CRIP1^+^ preHTC‐associated hub genes and can robustly discriminate OA from normal tissues, offering a candidate molecular diagnostic tool that requires further validation in future studies. These findings establish CRIP1^+^ preHTC as an important mediator in OA pathogenesis and offer new insights for targeted therapy and early diagnosis.

## Author Contributions

**Zikang Xie:** conceptualization, data curation, formal analysis, funding acquisition, writing – original draft. **Yu Wang:** formal analysis, methodology, validation. **Jinzhu Liu:** investigation, software. **He Li:** data curation. **Huwei Bian:** resources. **Chonghao Gu:** conceptualization, supervision, writing – review and editing.

## Funding

This work was supported by the Jiangsu Association of Chinese Medicine (JSACM) (Grant ZXFZ2026077) and the Changzhou Municipal Talent Work Leading Group Office for the “14th Five‐Year Plan” High‐Level Health Personnel Training Program of Changzhou (Grant 2024CZBJ031).

## Disclosure

All authors have read and approved the final manuscript.

## Ethics Statement

This work is based exclusively on published or publicly available datasets. Therefore, no ethical approval or informed consent was required.

## Conflicts of Interest

The authors declare no conflicts of interest.

## Data Availability

The data that support the findings of this study are available from the corresponding author upon reasonable request.
